# Editorial for the Special Issue “Genetic Sight: Plant Traits during Postharvest”

**DOI:** 10.3390/cimb45040229

**Published:** 2023-04-18

**Authors:** Shimeles Tilahun

**Affiliations:** 1 Agriculture and Life Science Research Institute, Kangwon National University, Chuncheon 24341, Republic of Korea; shimeles@kangwon.ac.kr; 2 Department of Horticulture and Plant Sciences, Jimma University, Jimma 378, Ethiopia

Modern breeding alternatives are less costly and sustainable solutions to increase quality, resistance to biotic and abiotic stresses, and to reduce postharvest losses of crops. Omic technologies, namely genomics, transcriptomics, proteomics, metabolomics, and phenomics, provide an efficient way to develop better cultivars. With this regard, review of the established omics technologies for fodder quality improvement through the improvement of the forage nutrition quality, edible quality, and digestibility is included in this special issue [1]. Similarly, studies of genetic relationships among the family, genera or cultivars of crops through molecular markers could assist in developing resistant cultivars to diseases and pests [2], to select super-earliness QTLs for prevention of heat-induced drought stress [3], and assist in seed production through profiling the pattern of protein expression under different crossing periods [4]. 

As the scope of research has expanded, studies have increasingly focused on the molecular mechanisms regulating a specific trait. This special issue incorporated about the regulation of flower development by CYC-like genes with their different functions and phylogenetic relationships in plant groups [5], function of DUF26 domain-containing genes to regulate the submergence tolerance of wild rice [6], and LbPYLs was suggested as good candidates to enhance Lycium resistance to drought and hot environments [7].

Considering postharvest qualities, candidate genes of plum associated with pulp color, anthocyanin biosynthesis and flavonoid biosynthesis [8], anthocyanin biosynthesis related MYBs in highbush blueberry [9], the potential genetic loci controlling quality traits of melons [10], and genes responsible for softening and ripening in kiwifruit cultivars treated with ethylene [11] were reported. 

Generally, as this special issue belongs to the section “Molecular Plant Sciences” and considering the interest of authors to contribute in this special issue, the editorial team tried to observe the broad spectrum, and eleven research articles [2,3,4,6,7,8,9,10,11,12,13] and two reviews [1,5] were included.

Finally, the Guest Editor would like to thank all authors for their excellent contributions and the Editorial Board for their support.
